# Defining the Timing Window: Week- and Interval-Specific Effects of Antenatal Betamethasone in Late-Preterm Births

**DOI:** 10.3390/jcm15041605

**Published:** 2026-02-19

**Authors:** Karin Edut, Ella Segal, Miriam Lopian, Ariel Many, Shanny Kolp-Asis

**Affiliations:** 1 Department of Obstetrics and Gynecology, Mayanei Hayeshua Medical Center, Bnei Brak 51544, Israel; 2 Grey Faculty of Health Sciences and Medicine, Tel Aviv University, Tel Aviv 6997801, Israel

**Keywords:** antenatal corticosteroids, betamethasone, late-preterm birth, respiratory distress syndrome, respiratory morbidity, neonatal hypoglycemia

## Abstract

**Objectives:** To evaluate the association between antenatal betamethasone exposure and neonatal respiratory morbidity among late-preterm births. We further examined whether gestational age at delivery and the exposure-to-delivery interval modify this association. **Methods:** We conducted a retrospective cohort study of singleton live births at 34–36 + 6 weeks in a tertiary center (2011–2023). Betamethasone exposure was classified as none, early (<34 weeks), or late (34–36 + 6 weeks). Among exposed pregnancies, the interval from first dose to delivery was categorized as ≤7 or >7 days and evaluated separately at 34, 35, and 36 weeks. Primary outcomes were RDS and composite respiratory morbidity (RDS, TTN, or ≥3 days of respiratory support); neonatal hypoglycemia was secondary. Adjusted odds ratios were estimated using multivariable logistic regression including maternal age, parity, delivery mode, and birthweight. **Results**: The study included 2668 late-preterm infants, of whom 2356 (88.3%) were unexposed and 312 (11.7%) were exposed to antenatal corticosteroids (ACSs). Among exposed pregnancies, 138 (44.2%) received early ACS and 174 (55.8%) late ACS; 163 (52.2%) delivered ≤7 days and 149 (47.8%) >7 days after administration. Late ACS exposure was associated with lower odds of RDS (aOR 0.37, 95% CI 0.17–0.69) and composite respiratory morbidity (aOR 0.55, 95% CI 0.31–0.92), but with increased odds of neonatal hypoglycemia (aOR 2.72, 95% CI 1.26–5.31). Among pregnancies delivering at 34 weeks, exposure within ≤7 days was associated with a marked reduction in RDS (aOR 0.25, 95% CI 0.07–0.79; NNT ≈ 3), whereas no respiratory benefit was observed at 35 or 36 weeks or when the interval exceeded 7 days. **Conclusions**: Antenatal betamethasone exposure among late-preterm births was not uniformly associated with neonatal respiratory outcomes, with associations varying by gestational age at delivery and the exposure-to-delivery interval. These findings may be interpreted in the context of potential respiratory benefit alongside accompanying metabolic risk, with exploratory analyses suggesting a more pronounced signal among deliveries at 34 weeks within ≤7 days.

## 1. Introduction

Reducing respiratory morbidity among late-preterm infants remains a major unmet clinical need, as this group constitutes the largest proportion of preterm births yet continues to experience substantial short- and long-term complications. Late-preterm infants, defined as those born between 34 + 0 and 36 + 6 weeks’ gestation, represent roughly 70% of all preterm births and up to 8% of all deliveries [1,2]. Although their outcomes are often assumed to approximate those of term neonates, late-preterm infants have higher rates of respiratory morbidity, hypoglycemia, temperature instability, and NICU admission [3,4]. Respiratory complications, principally transient tachypnea of the newborn (TTN) and respiratory distress syndrome (RDS), remain the leading drivers of short-term morbidity and resource use [1,2,3].

Physiologically, this vulnerability reflects a developmental stage in which both surfactant production and alveolar-fluid clearance remain incomplete. Antenatal corticosteroids (ACSs) cross the placenta and act on both pathways: they induce surfactant-protein gene expression and phospholipid biosynthesis and enhance ENaC and aquaporin expression, thereby improving compliance and stabilizing alveoli to facilitate gas exchange [5,6,7,8]. Glucocorticoids may also attenuate perinatal inflammatory and oxidative injury and decrease pulmonary-vascular permeability [5].

As suggested by Wang et al., respiratory distress in late-preterm and term neonates arises from mechanisms that differ from those in early-preterm infants, with reduced contribution from classic surfactant deficiency and greater involvement of impaired ENaC-mediated fluid clearance and perinatal factors such as cesarean delivery and inflammation [9]. These etiologic differences have been cited as one reason why prior late-preterm trials described benefits that were more prominent for TTN than for RDS, although the extent to which steroid responsiveness varies by gestational age remains uncertain. Glucocorticoid-related alterations in maternal-fetal glucose-insulin dynamics further provide a plausible mechanism for increased neonatal hypoglycemia [10]. Collectively, these considerations underscore the need to clarify the magnitude and mechanisms of ACS effects within the late-preterm window.

Following the ALPS randomized trial [11], ACOG and SMFM recommended a single course of betamethasone for individuals at risk of late-preterm delivery [12]. In ALPS, betamethasone reduced composite neonatal respiratory morbidity but increased neonatal hypoglycemia by approximately 60 percent. The respiratory benefit was driven primarily by TTN, with no significant reduction in RDS or mortality [11]. There was no clear breakdown of the benefits of ACS according to gestational age. Subsequent systematic reviews and multicenter RCTs have yielded mixed results: some confirm modest reductions in respiratory morbidity (particularly TTN), whereas others show no benefit for RDS or survival and raise concerns about maternal infectious morbidity and potential longer-term neurodevelopmental effects when exposure is followed by delivery at term [10,13]. These collective findings likely reflect, in part, heterogeneity in how exposure is defined, by gestational week, by administration-to-delivery interval, or by both.

Real-world cohorts further highlight timing as a determinant of efficacy and safety. In a dedicated late-preterm interval study, neonates delivered within the first 48 h after ACS administration had higher rates of TTN, RDS, and neonatal hypoglycemia compared with those delivered 2–7 days after exposure [14]. Consistently, latency analyses demonstrate that steroid-related hypoglycemia occurs within a broad 12–71 h window after administration, with peak incidence between 24 and 47 h [15]. Notably, a recent systematic review and meta-analysis of late-preterm randomized trials, including ALPS, demonstrated that these studies classified ACS exposure by gestational age at administration rather than at delivery, resulting in substantial misclassification because many steroid-exposed participants ultimately delivered at term [10]. Collectively, these data establish biological plausibility yet leave unresolved which subgroups truly benefit, at what gestational week, and within what interval, and whether TTN-focused benefits meaningfully extend to RDS.

Very few studies have focused specifically on pregnancies that both received antenatal corticosteroids and delivered within the late-preterm window [16]. We aimed to evaluate whether antenatal betamethasone administered in late-preterm pregnancies is associated with neonatal respiratory morbidity, including RDS, and whether these associations are modified by gestational age at delivery, by the exposure-to-delivery interval, and when analyzed separately at 34, 35, and 36 weeks. In a secondary analysis, we examined whether antenatal betamethasone is associated with neonatal hypoglycemia.

## 2. Materials and Methods

### 2.1. Study Design and Setting

This retrospective cohort study was conducted at a single tertiary medical center. The institutional perinatal database prospectively records all deliveries from November 2011 through May 2023. The study was approved by the Institutional Review Board, and informed consent was waived because of the nature of the study.

### 2.2. Study Population

All singleton live births delivered between 34 + 0 and 36 + 6 weeks’ gestation were eligible for inclusion. During the study period, 2751 infants met these gestational-age criteria. Exclusion criteria included intrauterine fetal demise; multiple gestations; chromosomal abnormalities diagnosed prenatally or postnatally; major structural congenital anomalies; and duplicate neonatal records. The final study cohort consisted of 2668 infants. Gestational age was assigned using the best obstetric estimate, based on first-trimester ultrasound corroborated by last menstrual period.

### 2.3. Exposure Definition

Antenatal corticosteroid exposure was defined by documented administration of betamethasone (Celestone) in obstetric and pharmacy records. Betamethasone was administered intramuscularly as two 12 mg doses given 24 h apart, constituting a single complete course.

Participants were classified into three mutually exclusive exposure groups:

(1) No betamethasone;
(2) Early exposure, defined as administration before 34 + 0 with delivery between 34 and 36 + 6 weeks;
(3) Late exposure, defined as administration between 34 + 0 and 36 + 6 weeks with delivery between 34 + 0 and 36 + 6 weeks.

Among all steroid-exposed pregnancies (early and late combined), the interval from the first betamethasone dose to delivery was classified as ≤7 days or >7 days, and these interval categories were also applied in week-specific analyses at 34, 35, and 36 weeks.

### 2.4. Outcome Definitions

Respiratory distress syndrome (RDS) was defined as respiratory distress shortly after birth accompanied by at least one of the following: chest radiograph consistent with surfactant deficiency; supplemental oxygen requirement greater than 40%; need for CPAP or mechanical ventilation attributed to surfactant deficiency; or administration of surfactant.

Transient tachypnea of the newborn (TTN) was defined by early-onset tachypnea, radiographic findings consistent with retained lung fluid, and resolution within 72 h. Respiratory support was defined as CPAP, high-flow nasal cannula, or mechanical ventilation for at least three consecutive days.

Composite respiratory morbidity was defined as the presence of RDS, TTN, or need for respiratory support for three or more consecutive days. Neonatal hypoglycemia was defined as blood glucose <45 mg/dL on two or more measurements within the first 24 h of life.

### 2.5. Statistical Analysis

Analyses were conducted using R version 4.4.1. Continuous variables were presented as mean ± standard deviation. Comparisons across three exposure groups were performed using one-way ANOVA. When comparisons involved two groups only, Welch’s two-sample *t*-test was used. Categorical variables were presented as *n* (%) and were compared using the chi-square test or Fisher’s exact test, as appropriate.

Crude odds ratios were estimated using univariable logistic regression. Multivariable logistic regression models were used to estimate adjusted odds ratios for respiratory distress syndrome, composite respiratory morbidity, and neonatal hypoglycemia. All adjusted models included maternal age, parity group, mode of delivery, and birth weight. Model fit was assessed using the Hosmer–Lemeshow goodness-of-fit test. A two-sided *p*-value < 0.05 was considered statistically significant.

## 3. Results

The study included 2668 infants born at 34–36 + 6 weeks. Of these, 2356 were included in the no-ACS group and 312 in the ACS-exposed group (Table 1 and Table 2). Among the ACS-exposed pregnancies, 138 received early exposure and 174 received late exposure.

Baseline maternal characteristics are summarized in Table 1, and Maternal and neonatal characteristics at delivery are summarized in Table 2. Gestational age at corticosteroid exposure differed between the early and late ACS groups, and gestational age at birth differed across the three comparison groups. Differences in birth weight and in the proportion of infants weighing <2500 g were also observed across the groups.

A difference in mode of delivery was noted, with a higher proportion of cesarean delivery in the early ACS group. PPROM rates were low and did not differ significantly between groups, and rates of gestational or pregestational diabetes were similar between the early and late ACS groups.

Within the 312 ACS-exposed pregnancies, 163 delivered within ≤7 days and 149 delivered >7 days after administration (Table 3). Gestational age at corticosteroid administration differed between the two interval groups. Differences in mode of delivery were observed, with a higher proportion of cesarean delivery among pregnancies delivering >7 days after ACS.

Univariable and multivariable logistic regression analyses were performed. Multivariable models were adjusted for maternal age, parity group, mode of delivery, and birthweight. Crude and adjusted odds ratios are presented in Table 4.

Neonatal outcomes differed across exposure categories. Early exposure (<34 weeks) did not show differences in RDS or composite respiratory morbidity compared with no exposure in adjusted analyses. Late exposure (34–36 + 6 weeks) was associated with lower adjusted odds of RDS and composite respiratory morbidity. These adjusted associations corresponded to an approximate number needed to treat (NNT) of 14–20. Late exposure was also associated with higher adjusted odds of neonatal hypoglycemia, with an estimated number needed to harm (NNH) of approximately 26.

Among exposed pregnancies, the interval from first dose to delivery showed gestational-age-related differences (Table 5). At 34 weeks, delivery within ≤7 days was associated with lower adjusted odds of RDS, corresponding to an approximate NNT of 3. A similar pattern was not observed at 35 or 36 weeks.

## 4. Discussion

### 4.1. Principal Findings

Our study suggests that the association between antenatal corticosteroid exposure in late-preterm birth depended jointly on the timing of exposure and the timing of delivery.

Early exposure did not demonstrate respiratory benefit. In contrast, late exposure was associated with lower rates of RDS and composite respiratory morbidity but with higher rates of neonatal hypoglycemia. Importantly, when analyses were stratified by gestational week and exposure-to-delivery interval, the most pronounced association was observed among pregnancies delivering at 34 weeks within ≤7 days of steroid administration. No measurable respiratory benefit was observed at 35–36 weeks or when the exposure-to-delivery interval exceeded this window. This interpretation is supported by the week-stratified analytic design, in which gestational age at delivery was held constant. However, these findings should be interpreted with caution, as causal attribution to the exposure-to-delivery interval alone is limited; timing of corticosteroid administration may partly reflect underlying clinical trajectories that are not fully captured by measured covariates.

Consistent with these findings, late-preterm respiratory vulnerability is not uniform across 34–36 weeks. Large epidemiologic datasets demonstrate a sharp decline in true RDS across this interval-decreasing from approximately 10% at 34 weeks to <3% at 36 weeks, and to 0.3% at term- with a nearly 40-fold difference in odds between 34 and 39 weeks [2]. This gestational-age gradient is biologically plausible and may be consistent with greater steroid responsiveness earlier in the late-preterm period, when surfactant immaturity still predominates, and provides a physiological basis for the week-specific pattern observed in our cohort.

Several recent analyses have attempted to clarify the relationship between timing of exposure, timing of delivery, and specific respiratory outcomes in the late-preterm period. Interval-focused cohorts, including the study by Gulersen et al. and others, identified a discrete 2–7-day window in which ACS appears most effective, with diminished benefit outside this interval [14,17]. However, these studies did not stratify outcomes by gestational week, and a substantial proportion of included neonates ultimately delivered at or near term. This lack of week-specific resolution limits comparability, as TTN predominated in these heterogeneous populations and may have masked steroid-responsive physiology present at earlier late-preterm gestations.

Similarly, the findings by Lau et al., which showed increased RDS when delivery occurred >7 days after dexamethasone exposure, further support the importance of interval-dependent efficacy; yet their broad gestational range (23 + 5–36 + 6 weeks) and use of dexamethasone instead of betamethasone restrict direct applicability to the late-preterm window [17]. Thus, while their findings reinforce the importance of timing, they do not clarify whether the respiratory response varies across 34–36 weeks.

Bulut et al. evaluated late-preterm pregnancies who received betamethasone and delivered within the same gestational window, and reported lower rates of RDS, TTN, and NICU admission among exposed neonates [18]. While directionally consistent with our observations, their reliance on unadjusted comparisons and the absence of week-specific stratification limit the ability to discern whether benefit varies across 34–36 weeks or by exposure–delivery interval. In contrast, our analysis incorporated stratification by week of delivery, exposure-to-delivery interval, and adjustment for selected clinical covariates, allowing a more detailed, exploratory assessment of these effects.

Finally, the most recent Cochrane review summarizes the established physiological mechanisms by which antenatal corticosteroids enhance fetal lung maturation and reduce RDS across the preterm spectrum [19]. However, the review does not provide late-preterm-specific subgroup analyses, limiting its direct applicability to the 34–36-week window.

The Antenatal Late Preterm Steroids (ALPS) trial remains the pivotal randomized evidence guiding ACS use at 34–36 weeks [11]. ALPS demonstrated a reduction in its composite respiratory outcome, driven predominantly by decreases in TTN and early respiratory support, with no significant reduction in RDS. Importantly, the median exposure-to-delivery interval was 33 h (IQR 15.2–111.6), placing most participants within a physiologically optimal window for steroid effect. At the same time, approximately 16% of infants delivered at ≥37 weeks, limiting the ability of the trial to isolate true late-preterm physiology. ALPS did not perform week-specific analyses, preventing evaluation of whether the respiratory benefits differed across 34, 35, and 36 weeks. Consequently, while ALPS established overall short-term respiratory benefit for late-preterm pregnancies, it could not determine whether a steroid-responsive RDS phenotype persists specifically at 34 weeks. Our findings add to this body of evidence by suggesting a week-specific pattern, with a more pronounced, exploratory respiratory signal observed among deliveries at 34 weeks in close proximity to steroid administration.

By contrast, the recent meta-analysis by Zullo et al. did not demonstrate a reduction in RDS with late-preterm ACS, identifying benefit only in measures of early respiratory support [10]. This discrepancy likely reflects three key methodological factors: the included trials did not stratify outcomes by gestational week; substantial proportions of near-term births were pooled with late-preterm deliveries, diluting steroid-responsive RDS phenotypes; and exposure-to-delivery intervals were not evaluated as effect modifiers [10]. Together, these limitations explain the absence of an RDS effect in the pooled analysis and underscore the importance of week- and interval-specific assessment, as explored in our cohort.

In our cohort, antenatal corticosteroid exposure in late-preterm pregnancies was associated with a significantly increased risk of neonatal hypoglycemia (Table 4). Week-specific interval analyses were underpowered; however, when all gestational weeks were pooled, infants delivered within seven days of ACS administration showed only a non-significant trend toward higher hypoglycemia rates (6.1% vs. 4.0%).

These findings are consistent with prior evidence. The ALPS randomized trial demonstrated a higher incidence of hypoglycemia among ACS-exposed neonates (24% vs. 15%), corresponding to an exposure window during which maternal hyperglycemia and fetal hyperinsulinemia are most likely to occur [11]. Similarly, McElwee et al. reported that hypoglycemia risk peaked when delivery occurred 12–47 h after ACS and declined thereafter, supporting a time-dependent effect [15].

By contrast, a recent meta-analysis of seven randomized trials did not show an overall increase in hypoglycemia among ACS-exposed late-preterm infants [10]. This discrepancy likely reflects heterogeneity in glucose-monitoring protocols, variation in diagnostic thresholds, and the lack of exposure–delivery interval stratification across included studies [10]. In our cohort, all diabetic women were hospitalized with tight glycemic control, minimizing the impact of corticosteroids on glucose control.

Although ACS-associated hypoglycemia is typically transient, the late-preterm period represents a critical developmental window characterized by rapid gains in gray- and white-matter volume [20,21]. Prior literature suggests that even asymptomatic neonatal hypoglycemia may be associated with white-matter vulnerability and later neurodevelopmental impairment [22].

Beyond short-term outcomes, the long-term safety profile of late-preterm corticosteroids has been strengthened by the recent ALPS follow-up study, which evaluated more than 900 children at 6–7 years of age using validated cognitive, behavioral, and motor assessments. Despite the higher rates of neonatal hypoglycemia observed in the steroid group in the original trial, no differences were identified in general cognitive ability, behavioral outcomes, or autistic traits. These findings provide important reassurance that the short-term respiratory benefits of ACS—particularly when exposure occurs within an effective interval—are unlikely to be offset by adverse neurodevelopmental sequelae. However, the study’s participation rate and reliance on standardized testing underscore the need for continued long-term surveillance, especially for infants delivering shortly after ACS exposure [23].

Strengths of this study include its large, well-characterized cohort of late-preterm singleton births, strictly limited to pregnancies that delivered between 34 + 0 and 36 + 6 weeks, allowing a focused assessment of the true physiological steroid response in this gestational window without dilution by term deliveries. Second, the combined stratification by gestational week and exposure-to-delivery interval highlighted a more pronounced, exploratory respiratory signal among deliveries at 34 weeks within ≤7 days of exposure, specifically reflected in reduced RDS. Finally, outcomes were rigorously defined using clinically relevant criteria, and multivariable models adjusting for selected obstetric and neonatal covariates allowed clinically meaningful interpretation of the findings, including numbers needed to treat and harm.

### 4.2. Limitations

It is a retrospective, single-center study, which raises the possibility of residual and unmeasured confounding despite adjustment for major clinical covariates. Week- and interval-specific subgroup analyses were limited by sample size, and categorizing exposure-to-delivery interval as ≤7 vs. >7 days may not fully capture finer temporal gradients in corticosteroid responsiveness. Stratified analyses by labor status were limited by the small number of patients without a trial of labor (elective cesarean delivery or cesarean delivery without labor onset), resulting in low statistical power and requiring cautious interpretation of this subgroup.

The diagnosis of neonatal hypoglycemia may be influenced by inherent variation in early glucose-screening patterns despite uniform institutional protocols. Finally, the study focused on short-term respiratory and metabolic outcomes, and longer-term respiratory or neurodevelopmental effects could not be assessed.

Across the late-preterm period, the magnitude of corticosteroid effect varied according to both gestational maturity and proximity to delivery. A more pronounced, exploratory respiratory signal was observed earlier in this gestational range and when exposure occurred in closer temporal proximity to birth, consistent with physiologic timing of surfactant responsiveness. Pregnancies exposed before 34 weeks but delivering later showed no clear respiratory advantage or harm, reflecting the limited relevance of remote exposure. In contrast, among pregnancies delivering at 35–36 weeks, respiratory outcomes did not differ meaningfully by exposure status; however, subgroup size limits the ability to exclude smaller, more modest effects that may still be clinically relevant. Corticosteroid exposure was associated with increased neonatal hypoglycemia in the overall late-preterm cohort, while week- and interval-specific estimates were insufficiently precise to determine whether this risk varies meaningfully across 34–36 weeks. Taken together, these findings suggest that corticosteroid effectiveness in late-preterm birth may reflect an interplay between gestational age and timing, with associations appearing stronger when biological maturity and exposure-to-delivery timing were well aligned.

## 5. Conclusions

Antenatal betamethasone exposure among late-preterm births was not uniformly associated with neonatal respiratory outcomes, with associations varying by gestational age at delivery and the exposure-to-delivery interval. These findings may be interpreted in the context of potential respiratory benefit alongside accompanying metabolic risk, with exploratory analyses suggesting a more pronounced signal among deliveries at 34 weeks within ≤7 days.

## Figures and Tables

**Table 1:** Maternal baseline characteristics by antenatal ACS exposure group.

Characteristic	No ACS	Early ACS	Late ACS	*p*-Value
Total, n (%)	2356 (88.3%)	138 (5.2%)	174 (6.5%)	
Maternal age, mean (SD), y	28.6 (6.27)	29.0 (6.28)	28.9 (6.21)	0.539
Maternal age categories, n (%), years				
<25	770 (32.7%)	42 (30.4%)	52 (29.9%)	0.66
25–34	1125 (47.7%)	65 (47.1%)	91 (52.3%)	0.499
35–39	295 (12.5%)	21 (15.2%)	17 (9.7%)	0.346
≥40	162 (6.9%)	10 (7.2%)	14 (8.0%)	0.836
Parity, n (%)				
Nulliparous	551 (23.4%)	40 (29.0%)	47 (27.0%)	0.199
Multiparous (1–5 births)	1469 (62.4%)	76 (55.1%)	104 (59.8%)	0.197
Grandmultiparous (≥6 births)	335 (14.2%)	22 (15.9%)	23 (13.2%)	0.788
BMI, mean (SD), kg/m^2^	26.27 (5.4)	23.89 (4.6)	27.14 (6.1)	0.201
Any hypertensive disorder, n (%)	137 (5.8%)	9 (6.5%)	18 (10.3%)	0.055
Gestational or pregestational diabetes, n (%)	243 (10.3%)	12 (8.7%)	13 (7.5%)	0.418
PPROM n (%)	58 (2.5%)	5 (3.6%)	5 (2.9%)	0.675
Gestational age at ACS exposure, mean (SD), wk		30.61 (2.7)	35.45 (0.7)	*p* < 0.001
Gestational age at ACS exposure, n (%)				—
<30 weeks	—	45 (32.6%)	—	
30–33 weeks	—	93 (67.4%)	—	
34 weeks	—	—	52 (29.9%)	
35 weeks	—	—	77 (44.3%)	
36 weeks	—	—	45 (25.9%)	

ACS = antenatal corticosteroids; Early ACS = administration <34 + 0 weeks; Late ACS = administration 34 + 0–36 + 6 weeks; PPROM = preterm prelabor rupture of membranes; BMI = body mass index; SD = standard deviation. Gestational age at ACS exposure is shown for the ACS groups only.

**Table 2:** Maternal and neonatal characteristics at delivery by antenatal ACS exposure group.

Characteristic	No ACS	Early ACS	Late ACS	*p*-Value
Total, n (%)		2356 (88.3%)	138 (5.2%)	174 (6.5%)
Mode of delivery, n (%)				
Vaginal delivery	1644 (69.8%)	65 (47.1%)	106 (60.9%)	*p* < 0.001
Cesarean delivery	604 (25.6%)	65 (47.1%)	61 (35.1%)	*p* < 0.001
Operative vaginal or Breech delivery	108 (4.6%)	8 (5.8%)	7 (4.0%)	0.747
Gestational age at birth, mean (SD), wk ᵃ	35.97 (0.8)	35.51 (0.96)	35.87 (0.67)	*p* < 0.001
Gestational age at birth, n (%)				
34 weeks	307 (13.0%)	46 (33.3%)	21 (12.1%)	*p* < 0.001
35 weeks	588 (25.0%)	29 (21.0%)	61 (35.1%)	0.006
36 weeks	1461 (62.0%)	63 (45.7%)	92 (52.9%)	*p* < 0.001
Male, n (%)	1325 (56.2%)	83 (60.1%)	98 (56.3%)	0.670
Birth weight, mean (SD), g	2595.89 (446.92)	2426.57 (472.81)	2513.84 (456.03)	*p* < 0.001
Birth weight <2500 g n (%)	928 (39.4%)	74 (53.6%)	86 (49.4%)	*p* < 0.001

ACS = antenatal corticosteroids; Early ACS = administration <34 + 0 weeks; Late ACS = administration 34 + 0–36 + 6 weeks; SD = standard deviation. ᵃ Based on group means, the difference in mean gestational age at birth between the early ACS and no-ACS groups was approximately 0.47 weeks (≈3.3 days).

**Table 3:** Maternal and neonatal characteristics of ACS-exposed pregnancies by exposure-to-delivery interval.

Characteristic	≤7 n (%)	>7 n (%)	*p*-Value
Total, n (%)	163 (52.2%)	149 (47.8%)	
Maternal age, mean (SD), y	28.97 (6.14)	28.99 (6.35)	0.973
Maternal age categories, n (%)			
<25	46 (28.2%)	48 (32.2%)	0.4425
25–34	87 (53.4%)	69 (46.3%)	0.2125
35–39	18 (11.0%)	20 (13.4%)	0.5209
≥40	12 (7.4%)	12 (8.1%)	0.8188
Parity, n (%)			
Nulliparous	43 (26.4%)	44 (29.5%)	0.5354
Multiparous (1–5 births)	99 (60.7%)	81 (54.4%)	0.255
Grandmultiparous (≥6 births)	21 (12.9%)	24 (16.1%)	0.4181
Any hypertensive disorder, n (%)	16 (9.8%)	11 (7.4%)	0.445
Gestational or pregestational diabetes, n (%)	13 (8.0%)	12 (8.1%)	0.9797
PPROM n (%)	7 (4.3%)	3 (2.0%)	0.3408
Mode of delivery, n (%)			
Vaginal delivery	101 (62.0%)	70 (47.0%)	0.0079
Cesarean delivery	55 (33.7%)	71 (47.7%)	0.0124
Operative vaginal or Breech delivery	7 (4.3%)	8 (5.4%)	0.6576
Gestational age at birth, mean (SD), wk	35.74 (0.78)	35.67 (0.90)	0.496
Gestational age at birth, n (%)			
34 weeks	29 (17.8%)	38 (25.5%)	0.0975
35 weeks	56 (34.4%)	34 (22.8%)	0.0247
36 weeks	78 (47.9%)	77 (51.7%)	0.4997
Male, n (%)	93 (57.1%)	88 (59.1%)	0.72
Birth weight, mean (SD), g	2494.62 (453.33)	2453.91 (477.65)	0.442
Birth weight <2500 g, n (%)	84 (51.5%)	76 (51.0%)	0.881
Gestational age at ACS exposure, mean (SD), wk	35.46 (0.78)	30.95 (2.88)	*p* < 0.001
Gestational age at ACS exposure, n (%)			
<30 weeks	—	45 (30.2%)	—
30–33 weeks	8 (4.9%)	84 (56.37%)	*p* < 0.001
34 weeks	35 (21.5%)	17 (11.4%)	0.0172
35 weeks	75 (46.0%)	2 (1.3%)	*p* < 0.001
36 weeks	45 (27.6%)	—	—

Exposure-to-delivery interval was defined as the time from the first dose of betamethasone to delivery and categorized as ≤7 days or >7 days. ACS = antenatal corticosteroids; PPROM = preterm prelabor rupture of membranes; SD = standard deviation.

**Table 4:** Neonatal outcomes by antenatal ACS exposure. Reference: No ACS.

Outcome	Comparison	Events/Total	Crude OR (95% CI)	*p*-Value	Adjusted OR (95% CI)	*p*-Value	NNT/NNH
RDS	Early ACS	25/138	1.61 (1.01–2.49)	0.037	1.33 (0.82–2.09)	0.224	NNH 16.51
RDS	Late ACS	9/174	0.40 (0.19–0.74)	0.008	0.37 (0.17–0.69)	0.005	NNT 14.52
Respiratory morbidity	Early ACS	27/138	1.53 (0.97–2.33)	0.058	1.25 (0.79–1.94)	0.326	NNH 17.22
Respiratory morbidity	Late ACS	15/174	0.59 (0.33–0.98)	0.058	0.55 (0.31–0.92)	0.034	NNT 19.47
Hypoglycemia	Early ACS	6/138	2.28 (0.86–5.04)	0.063	1.62 (0.60–3.68)	0.287	NNH 41.76
Hypoglycemia	Late ACS	10/174	3.06 (1.44–5.93)	0.002	2.72 (1.26–5.31)	0.006	NNH 26.36

Crude odds ratios were estimated using univariable logistic regression. Adjusted odds ratios were estimated using multivariable logistic regression models adjusted for maternal age, parity, mode of delivery, and birthweight. NNT = number needed to treat; NNH = number needed to harm; OR = odds ratio; CI = confidence interval; ACS = antenatal corticosteroids; RDS = respiratory distress syndrome; composite respiratory morbidity = RDS, transient tachypnea of the newborn (TTN), or ≥3 days of respiratory support.

**Table 5:** Neonatal outcomes by exposure-to-delivery interval, stratified by gestational age at delivery (weekly categories). Reference: >7 days.

Gestational Age at Delivery	Outcome	Exposure Interval	Events/Total (%)	aOR (95% CI)	*p*-Value	NNT/NNH
34 + 0–34 + 6	RDS	>7 days	18/38 (47.4%)	1.00	—	—
		≤7 days	5/29 (17.2%)	0.25 (0.07–0.79)	0.023	NNT 3.32
35 + 0–35 + 6	RDS	>7 days	3/34 (8.8%)	1.00	—	—
		≤7 days	3/56 (5.4%)	1.01 (0.15–8.18)	0.990	NNT 29.68
36 + 0–36 + 6	RDS	>7 days	3/77 (3.9%)	1.00	—	—
		≤7 days	2/78 (2.6%)	0.89 (0.11–6.06)	0.901	NNT 75.08
34 + 0–34 + 6	Respiratory morbidity	>7 days	19/38 (50.0%)	1.00	—	—
		≤7 days	8/29 (27.6%)	0.42 (0.13–1.25)	0.126	NNT 4.46
35 + 0–35 + 6	Respiratory morbidity	>7 days	5/34 (14.7%)	1.00	—	—
		≤7 days	4/56 (7.1%)	0.57 (0.12–2.68)	0.468	NNT 13.45
36 + 0–36 + 6	Respiratory morbidity	>7 days	3/77 (3.9%)	1.00	—	—
		≤7 days	3/78 (3.8%)	1.41 (0.23–8.74)	0.698	NNT 2002.00
34 + 0–34 + 6	Hypoglycemia	>7 days	2/38 (5.3%)	1.00	—	—
		≤7 days	7/29 (24.1%)	13.52 (2.19–144.88)	0.011	NNH 5.30
35 + 0–35 + 6	Hypoglycemia	>7 days	0/34 (0.0%)	1.00	—	—
		≤7 days	1/56 (1.8%)	Not calculable	0.999	NNH 55.00
36 + 0–36 + 6	Hypoglycemia	>7 days	4/77 (5.2%)	1.00	—	—
		≤7 days	2/78 (2.6%)	0.59 (0.07–3.54)	0.567	NNT 38.01

Adjusted odds ratios (aORs) were estimated using logistic regression models adjusted for maternal age, parity, mode of delivery, and birthweight. The exposure-to-delivery interval was defined as the time from the first dose of betamethasone to delivery and categorized as ≤7 days or >7 days. The reference group for all comparisons was exposure-to-delivery interval >7 days. NNT = number needed to treat; NNH = number needed to harm; CI = confidence interval; RDS = respiratory distress syndrome; composite respiratory morbidity includes RDS, transient tachypnea of the newborn (TTN), or ≥3 days of respiratory support.

## Data Availability

Data are available from the corresponding author upon reasonable request, due to privacy and ethical restrictions.

## Abbreviations

The following abbreviations are used in this manuscript:

ACS	antenatal corticosteroids
BMI	body mass index
RDS	respiratory distress syndrome
TTN	transient tachypnea of the newborn
PPROM	preterm premature rupture of membranes
OR	odds ratio
aOR	adjusted odds ratio
CI	confidence interval
NNT	number needed to treat
NNH	number needed to harm
